# Suicidal ideation, mental health services, and barriers to care among syringe services program clients who use opioids

**DOI:** 10.1371/journal.pone.0354745

**Published:** 2026-07-30

**Authors:** Kiran Paudel, Kamal Gautam, Sherry Pagoto, Ran Xu, Jeffrey A. Wickersham, Frederick L. Altice, Sandesh Bhusal, Md. Safaet Hossain Sujan, Michael Copenhaver, Chenglin Hong, Roman Shrestha

**Affiliations:** 1 Department of Allied Health Sciences, University of Connecticut, Storrs, Connecticut, United States of America; 2 Nepal Health Frontiers, Kathmandu, Nepal; 3 Section of Infectious Diseases, Department of Internal Medicine, Yale School of Medicine, New Haven, Connecticut, United States of America; 4 Yale School of Public Health, Department of Epidemiology of Microbial Diseases, New Haven, Connecticut, United States of America; 5 Center for Interdisciplinary Research on AIDS, Yale University, New Haven, Connecticut, United States of America; 6 School of Social Work, University of Connecticut, Connecticut, United States of America; University of Toronto, CANADA

## Abstract

**Introduction:**

People who use opioids (PWUO) experience a disproportionately high burden of suicidal risk. Syringe services programs (SSPs) are a critical point of contact for this population, providing low-threshold, harm-reduction services. However, gaps persist in understanding suicidal risk among individuals attending SSPs and the extent to which such risk is associated with mental health service utilization in this setting. This study aimed to assess the prevalence and specific risk factors for suicidal ideation (SI) in PWUO, as well as their use of and barriers to accessing mental health services.

**Methods:**

We conducted a cross-sectional survey between April and June 2024 among PWUO (N = 199) who were utilizing services in the SSP in New Haven, Connecticut. We used the ninth item from the Patient Health Questionnaire (PHQ-9) scale, which assesses passive thoughts of death and self-harm in the past two weeks, as a proxy measure for the SI. Additionally, participants reported their use of and barriers to accessing mental health services in the past six months. Bivariate and multivariable logistic regression were conducted to examine factors associated with SI.

**Results:**

Approximately 84% of participants met the criteria for opioid dependence, and more than two-thirds (71.9%) were currently receiving medication for opioid use disorder. Over one-third of the sample reported SI in the past two weeks (34.7%). Additionally, over one-third of participants (36.2%) reported unmet mental health needs, and 84.4% reported experiencing at least one barrier to accessing mental health services. Individuals with depressive symptoms (aOR: 5.4; 95% CI: 2.5–11.7) were more likely to report SI, while those who were aware of naloxone (aOR: 0.2; 95% CI: 0.1–0.6) were less likely to report SI in the past two weeks.

**Conclusion:**

Our study showed a high prevalence of SI among SSP clients who use opioids and a low use of mental health services due to access barriers. These findings support incorporating a brief suicide risk screening and referral options within SSPs and other settings serving PWUO and addressing key access issues like cost and transportation that can prevent timely linkage to mental health services.

## Introduction

The opioid epidemic has remained an alarming public health crisis over the last two decades, with the United States accounting for approximately 80% of total global opioid use [1–3]. In 2022, 76% of the 108,000 drug overdose deaths in the US involved an opioid, which means an average of 224 people died each day due to opioid overdoses [4]. That same year, suicide was the 11^th^ leading cause of death in the US, with over 49,000 deaths [5]. A growing body of evidence underscores the interdependence between illicit opioid use and suicide risk, driven by multi-level factors (factors operating across individuals and structural levels) such as co-occurring mental health issues, chronic pain, alcohol use disorder, homelessness, opioid dependence, and risk of overdose [6–8]. The combination of these two crises has led to a historic three-year decline in U.S. life expectancy, the first reversal of this kind in more than a century [9].

Individuals with opioid use are estimated to have suicide risk six times higher than the general populations [10]. Non-medical opioid use is consistently associated with increased odds of suicidal ideation [11]. Despite the high burden, they fall short in addressing psychological and contextual factors such as housing instability, HIV risk, and inadequate access to mental health care that contribute to suicidality [12,13].

Syringe services programs (SSPs) are a critical point of contact for people who use opioids (PWUO), which provide low-threshold, harm-reduction-oriented services [14]. Importantly, SSPs often engage individuals who are not connected to traditional healthcare systems, positioning them as trusted, community-based settings where unmet health and social needs can be identified [15]. Their non-judgmental, client-centered approach can reduce stigma and foster trust, making them uniquely suited to reach individuals who may otherwise avoid formal care [15]. However, despite this potential, integration of mental health and suicide prevention services within SSPs remains limited and inconsistent, often constrained by funding, staffing, and policy environments [16,17].

Regular screening for suicidal ideation (SI) for individuals at higher suicidal risk is essential, as early detection can facilitate timely referral, linkage to care, and prevention efforts before thoughts escalate into self-harm or death [18]. Despite this burden, there remains a significant lack of research on suicide screening and intervention within community-based harm reduction settings, such as SSPs. Furthermore, little is known regarding the mental services utilization and the barriers among PWUO experiencing suicidal ideation accessing SSPs. Such gaps exist in understanding suicide risk among PWUO and how multiple barriers affect access to mental health care.

To address this gap in the literature, we explored the prevalence of SI and examined the mental health service utilization and barriers to accessing care among adults who use opioids at the community-based SSP. Findings from this study can help to inform integrated, low barrier intervention that leverage SSPs as platforms for both harm reductions and suicide prevention.

## Methods

### Study design and study participants

A cross-sectional survey was conducted among 199 PWUO between April and June 2024 at the New Haven SSP, which provides primary healthcare and HIV prevention services to PWUO in the greater New Haven area, Connecticut. NHSSP operates as a harm reduction center providing syringe exchange and wraparound services to people who use drugs. It also has a mobile health care van that travels to multiple locations within New Haven to provide free and accessible services to PWUO and other vulnerable groups in the community [19]. Eligibility criteria of the study included: i) being 18 years or older; ii) self-reported heroin or other opioid drugs (illicit) use in the past 30 days; iii) able to read and speak English; and iv) currently residing in or around greater New Haven, Connecticut. This study was reported in accordance with the STROBE guidelines for cross-sectional studies (S1 Checklist).

### Study procedure

A purposive sampling technique was used to recruit participants through flyers, word of mouth, and direct referrals from the SSP staff. Trained research assistants conducted eligibility screening, enrollment, and interviewed participants in-person in a private room at the NHSSP. Written informed consent was obtained from the participants before starting the survey. Participants completed the survey in the private room at NHSSP, fixed site. The study took approximately 30 minutes to complete, and participants received $10 in compensation.

### Ethical considerations

The Institutional Review Board of Yale University (IRB Protocol ID: 2000035622) approved the study. Written informed consent was obtained from all participants upon proceeding with the survey. All the study procedures adhered to the ethical standards outlined in the Declaration of Helsinki and all participants provided informed consent.

### Measures

#### Outcome variable.

We used the ninth item from the Patient Health Questionnaire (PHQ-9) to measure SI. This item endorsed thoughts of death or self-harm in the past 2 weeks. It is recommended by the Joint Commission as a brief, self-report tool for suicide risk screening, and has been used as a proxy measure for suicidal ideation across different populations [20–22]. Participants responded on a 4-point Likert scale from ‘not at all’ to ‘nearly every day’ to the question, “O*ver the last 2 weeks,* *how often have you been bothered by the following problem: thoughts that you would be better off dead, or of hurting yourself in some someway?*” A response of ‘several days’, ‘more than half the days’, and ‘nearly every day’ was recorded as ‘Yes’ for suicidal ideation [23].

The independent variables included socio-demographic, behavioral, and health-related characteristics. The instruments used in this study have been previously used in comparable settings and similar research contexts [21,24–27].

### Socio-demographic variables

Participants were asked to provide their age, sex at birth, sexual orientation, race or ethnicity, educational status, employment status, relationship status, and current housing status in the last 12 months. Race and ethnicity were combined and recoded into three groups: “Non-Hispanic White”, “Hispanic”, and “Others” (Black or non-Hispanic, Asian, African American). Sexual orientation was assessed via a single self-report item. For analysis, responses were dichotomized into two categories: gay or lesbian and heterosexual. Similarly, educational status was categorized into three groups: “Less than high school”, “High school completed”, and “Bachelor’s degree and above.” Employment was coded as employed or unemployed, and relationship status was coded as single or partnered. Housing status was assessed using a single item asking whether participants had experienced homelessness in the last 12 months.

### General health-related characteristics

#### HIV status.

Participants’ HIV status was assessed with the question “*Have you ever been diagnosed with HIV in your lifetime*?” with a dichotomous response (Yes/No).

### Depressive symptoms

The PHQ-8 scale, which corresponds to the DSM-IV’s diagnostic criteria for major depressive disorder, was used to assess depressive symptoms [28]. Participants responded using a four-point Likert scale, ranging from “0 = not at all” to “4 = almost every day.” A cutoff score of ≥10 was used to determine moderate to severe depressive symptoms, as this threshold is a validated predictor of a major depressive disorder diagnosis [28–30].

### Substance use treatment

Participants were asked to indicate the type of drug treatment they were receiving, if any, at the time of the survey. Response options included methadone, buprenorphine, vivitrol (extended-release naltrexone), other treatment types, and none. For the analysis, these were combined into a single binary variable reflecting whether the participants were currently in treatment for opioid use.

### Mental health service utilization

Mental health service use in the past 6 months was assessed by asking: “*Have you seen a mental health professional in the last 6 months?*” (‘Yes’, ‘no, but I needed to, and ‘no, because I did not need to’). A follow-up question was asked to assess perceived barriers to accessing mental health care, including knowledge (unsure what to do to get professional care), stigma (concerned for what others might think, say, or do), transportation (difficulty with transport or travelling for treatment), expense (unable to afford the expenses that followed), and negative experiences (had bad experiences with professional care for mental health problems). Response options were four-point Likert scales ranging from ‘Not at all’ to ‘A lot’. We dichotomized the responses by categorizing ‘Not at all’ as ‘No” and all other options as “Yes”. These questions were adopted from previous studies [31,32].

### Substance use and treatment-related variables

The Alcohol Use Disorders Identification Test-Consumption (AUDIT-C) is a brief, validated screening tool for alcohol use and dependence (also known as alcohol misuse) and problematic drinking [33]. The potential AUDIT-C scores range from 0 to 12 points, with each item’s response alternatives worth 0–4 points. As recommended, AUDIT-C scores of at least 4 for males and at least 3 for females are coded as AUD [33,34].

Opioid dependence was assessed using the Rapid Opioid Dependence Screen (RODS), an 8-item yes/no questionnaire that identifies key features of opioid dependence [35]. The first question asks whether the participant had used any opioid in the last 12 months, such as heroin, methadone, buprenorphine, morphine, MS Contin, OxyContin, oxycodone, and other opioid analgesics. A “no” response to all drug types resulted in an immediate outcome of non-dependence. Participants who responded “yes” to any of the drugs were asked questions through 2–8. Participants who answered “yes” to three or more of the follow-up questions (items 2–8) were categorized as opioid dependent. The RODS has demonstrated strong psychometric properties such as internal consistency α = 0.92, sensitivity (.97), and specificity (.76), as established by Wickersham, J. and et.al [35].

Lifetime experience of non-fatal drug overdose was assessed by asking participants whether they had ever experienced a drug-related overdose in their lifetime (Yes/No). We also assessed participants’ awareness of naloxone as “before participating in this survey, have you ever heard of naloxone?” (Yes/No). Follow-up questions were asked about whether participants carry Naloxone or Narcan.

### Statistical analysis

Data were collected from Qualtrics and imported into StataCorp LLC version 18.0 for data cleaning and analysis. Descriptive statistics were employed to summarize the data, including frequencies and percentages for categorical variables and means with standard deviations for continuous variables. Chi-square tests and independent-samples t-tests were conducted for group comparisons of categorical and continuous independent variables, respectively. Bivariate analyses were performed to identify variables for inclusion in the adjusted regression analysis. Variables whose p-value is less than 0.1 were fitted into the multivariable logistic regression model [36].

The multivariate logistic regression was used to identify significant correlates of suicidal ideation. The test for multicollinearity (by calculating variance inflation factor scores for each predictor variable) revealed no evidence of multicollinearity (all values were less than 1.05) [36]. Adjusted odds ratios (aOR) were computed with a 95% confidence interval (CI), and statistical significance was defined as a p-value less than 0.05.

## Results

**Fig 1:**
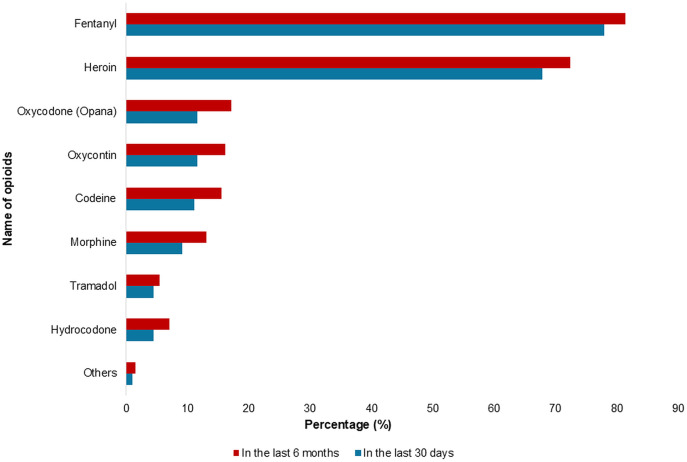
Opioid use in the last 6 months and 30 days.

Table 1 summarizes participant characteristics. Participants were on average 44.2 (± 10.2) years old and more than two-thirds (79.9%) were homeless. More than half (58.3%) reported moderate to severe depressive symptoms and 4.5% reported living with HIV. Over one-third of participants (34.7%) reported experiencing suicidal ideation. More than half of the participants (55.8%) reported a lifetime history of non-fatal drug overdose, and the majority (87.4%) had heard of naloxone. Approximately 84% of the participants met criteria for opioid dependence and more than two-thirds (71.9%) were currently receiving substance use treatment. The most commonly used opioids in the last 30 days were fentanyl (77.9%) and heroin (67.8%; see Fig 1).

**Table 1:** General Characteristics of the participants.

Characteristics	Number	Percentage
**Socio Demographic**		
Age (Years) (Mean ±SD)	44.2 ± 10.2	
Educational status		
Less than high school	16	8.1
High school completed	166	83.4
Bachelor’s and above	17	8.5
Sex at birth		
Male	131	65.8
Female	67	33.7
Others	01	0.5
Sexual orientation		
Gay or lesbians	24	24.1
Heterosexual	175	87.9
Relationship status		
Single	144	72.4
With partner	55	27.6
Ethnicity		
Hispanic	43	21.6
Non-Hispanic White	120	60.3
Others	36	18.1
Unemployed	183	92.0
Homelessness in the last 12 months	159	79.9
**General health-related characteristics**		
Living with HIV	9	4.5
Depressive symptoms (moderate to severe)	117	58.3
Suicidal ideation	69	34.7
Currently on substance use treatment	143	71.9
**Substance use-related characteristics**		
Alcohol use disorder	131	65.8
Opioid dependent	167	83.9
Ever experienced a non-fatal drug overdose	111	55.8
Heard of naloxone	174	87.4
Carry naloxone or Narcan	149	74.9

**Fig 2:**
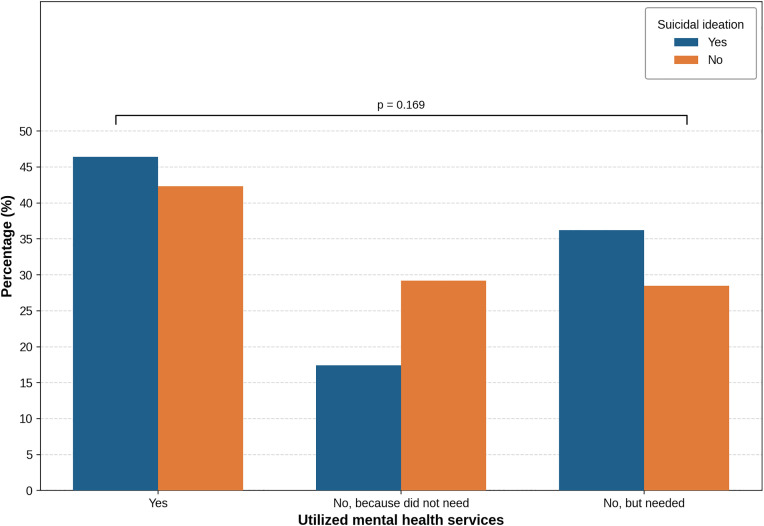
Mental health care services utilization in the last 6 months stratified by suicidal ideation.

Over one-third of the participants with SI (36.2%) reported that they needed mental health services but were not receiving them (see Fig 2). In contrast, nearly one in five (17.4%) of those with SI reported that they did not perceive a need for mental health care.

**Fig 3:**
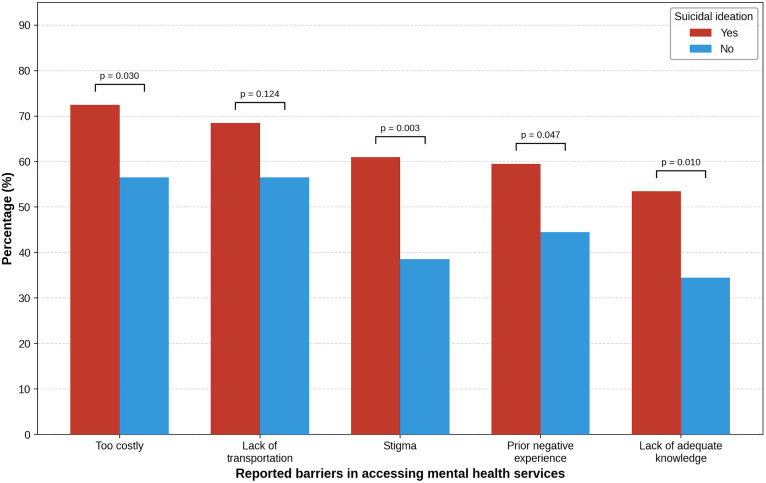
Perceived barriers to accessing mental health care stratified by suicidal ideation.

Among the participants who expressed concerns about accessing mental health services, financial constraint was the most commonly reported barrier (62.3%), followed by transportation challenges (60.8%). Most participants (84.4%) reported that they had at least one barrier to accessing mental health services. More than two-thirds (70.3%) had two or more barriers, and 17.1% faced all identified barriers (see Supplementary Figure 1). As shown in Fig 3, a higher proportion of individuals with SI, compared to those without SI, reported having experienced various barriers in accessing mental health services. See Table 2 for the bivariate analysis of SI among PWUO.

**Table 2:** Bivariate analysis of suicidal ideation among PWUO.

Variable	Suicidal Ideation		Unadjusted odds ratio (95% CI)	P value
	Yes (%)	No (%)		
Age (Years): Mean ± SD	44.3 ± 10.3	44.2 ± 10.2	1.0 (0.9-1.1)	0.6
Educational status				
Less than high school	8 (50.0)	8 (50.0)	1.8 (0.5-7.4)	0.5
High school completed	55 (33.1)	111 (66.9)	0.9 (0.3-2.6)	0.3
Bachelor’s and above	6 (35.3)	11 (64.7)	Ref	
Sexual Orientation				
Gay or lesbians	8 (33.3)	16 (66.7)	0.9 (0.4-2.3)	0.9
Heterosexual	61 (34.9)	114 (65.1)	Ref	
Ethnicity				
Hispanic	20 (46.5)	23 (53.5)	2.3 (0.9-5.8)	0.1
Non-Hispanic White	39 (32.5)	81 (67.5)	1.3 (0.5-2.9)	0.5
Others	10 (27.8)	26 (72.2)	Ref	
Relationship status				
Single	48 (33.3)	96 (66.7)	0.8 (0.4-1.5)	0.5
With partner	21 (38.2)	34 (61.8)	Ref	
Employment status				
Employed	5 (31.3)	11 (68.8)	0.8 (0.3-2.5)	0.7
Unemployed	64 (34.9)	119 (65.1)	Ref	
Homelessness *(last 12 months)*				
Yes	55 (34.6)	104 (65.4)	0.9 (0.5-1.8)	0.9
No	14 (35.0)	26 (65.0)	Ref	
Living with HIV				
Yes	5 (55.6)	4 (44.4)	2.5 (0.6-9.5)	0.2
No	64 (33.7)	126 (66.3)	Ref	
Depressive symptoms				
Moderate to severe	54 (46.5)	62 (53.5)	3.9 (2.0-7.7)	<0.001
None to mild	15 (18.1)	68 (81.9)	Ref	
Opioid dependent				
Yes	62 (37.1)	105 (62.9)	2.1 (0.9-5.2)	0.09
No	7 (21.9)	25 (78.1)	Ref	
Currently on substance use treatment				
Yes	51 (35.7)	92 (64.3)	1.2 (0.6-2.3)	0.6
No	18 (32.1)	38 (67.9)	Ref	
Experienced overdose *(Ever)*				
Yes	43 (38.7)	68 (61.3)	1.5 (0.8-2.7)	0.2
No	26 (29.6)	62 (70.4)	Ref	
Heard of naloxone				
Yes	56 (32.2)	118 (67.8)	0.4 (0.2-1.0)	0.05
No	13 (52.0)	12 (48.0)	Ref	
Carry naloxone or Narcan				
Yes	48 (32.2)	101 (67.8)	0.7 (0.3-1.3)	0.2
No	21 (42.0)	29 (58.0)	Ref	
Alcohol use disorder				
Yes	47 (35.9)	84 (64.1)	1.2 (0.6-2.2)	0.6
0 No	22 (32.4)	46 (67.6)	Ref	

### Factors associated with suicidal ideation

Participants who reported moderate to severe depressive symptoms (aOR: 5.8; 95% CI: 2.7–12.4) had higher odds of experiencing SI compared to those with none to mild depressive symptoms. Interestingly, those who were aware of naloxone (aOR: 0.3; 95% CI: 0.1–0.6) were less likely to have experienced SI (see Table 3).

**Table 3:** Factors associated with suicidal ideation.

Variables	Unadjusted Odds ratio (95% CI)	Adjusted Odds ratio (95% CI)
Depressive symptoms		
Moderate to severe	3.9 (2.0-7.7)	4.9 (2.3-10.2)
None to mild	Ref	Ref
Heard of naloxone		
Yes	0.4 (0.2-1.0)	0.2 (0.1-0.6)
No	Ref	Ref
Opioid dependent		
Yes	2.1 (0.9-5.2)	1.6 (0.6-4.2)
No	Ref	Ref

## Discussion

Our findings revealed a high burden of suicidal ideation (34.7%) among SSP clients who use opioids. The elevated risk could be driven by a multilevel factor such as biological, psychological, social and structural factors including neurobiological effects of opioid use, co-occurring mental illness, social adversity (homelessness, food insecurity, unemployment) and genetic vulnerability [37–39]. Despite this high burden, SSPs more focus primarily on harm reduction and lacks suicide prevention services such as screening, and linkage to care [40]. National data show rising SI among PWUO without a corresponding increase in mental health service use, underscoring missed opportunities in routine touchpoints like SSPs [10]. To address the current lack of suicide screening and referral services at SSPs, our findings underscore the need for routine assessment of suicide risk at key healthcare touchpoints for PWUO.

Our study revealed that PWUO experiencing SI encounter more barriers to mental health services and exhibit lower utilization rates than the general population, despite having the greatest need, which is in line with prior findings [10,41]. Low rates of mental health care utilization may be due to PWUO avoiding healthcare settings for fear of legal consequences due to illegal drugs or judgmental treatment from healthcare providers [12]. Prevalent barriers to accessing mental health care, as shown in our findings, may worsen the suicide risk by delaying or preventing care, particularly among those experiencing SI [10]. Among individuals accessing SSPs, many of whom already distrust emergency and legal systems [12], SI screening may be perceived as a trigger for involuntary transport or police involvement for emergency evaluation, thereby discouraging engagement with services [42]. To address this, screening protocols could be co-designed with participants and embedded within the SSPs. Furthermore, these efforts could be supported by broader policy changes that reduce the role of law enforcement in responding to mental health and opioid crises.

Interestingly, unlike other prior studies [43–45], our study found that PWUO who had heard of naloxone were less likely to experience SI. This association may reflect the indirect benefits of engagement with harm reduction services and supportive care environments. Individuals who are aware of such harm reduction services, such as overdose prevention using naloxone, are more likely to be connected to community-based resources, such as SSPs, where they receive education, social support, and non-judgmental interaction that can mitigate feelings of isolation and distress [46,47]. Participation in naloxone training programs in these settings may also foster a sense of agency and self-efficacy by equipping individuals with the skills to prevent overdoses and help others in crisis [48]. Together, these experiences may counteract feelings of hopelessness by reinforcing a sense of purpose, competence and connection [46].

Not surprisingly, we found that individuals with depressive symptoms had higher odds of experiencing SI, which is in line with findings from previous studies [26,49,50]. More than half (58.8%) of our sample had moderate to severe depressive symptoms, potentially reflecting the cumulative impact of chronic pain, social isolation, withdrawal symptoms and related stressors. All these factors may elevate the feeling of hopelessness and psychological distress, contributing to SI [51]. Moreover, the co-occurrence of depressive symptoms and opioid use exacerbates the SI risk [10]. Therefore, these findings highlight the importance of integrated mental health care within healthcare treatment settings that serve this vulnerable population.

### Limitations

This study has certain limitations that need to be acknowledged. First, the generalizability of our findings is limited, as the sample was drawn from a single county in Connecticut with low sociodemographic diversity. However, the sample included a substantial proportion of sexual minority individuals (gay or lesbian: 24.1%), considerably exceeding general population estimates, and meaningful representation of Hispanic (21.6%) which strengthens the relevance of findings for these underserved populations. Second, convenience sampling from one SSP introduced selection bias favoring individuals already linked to existing services. Third, because of the cross-sectional design, causal inferences or temporality cannot be made, especially because the outcome window (2 weeks) differs from the service window (6 months), and longitudinal studies are warranted to explore temporal relationships. Four, although we assessed participants’ awareness of naloxone, we did not assess their history of use, their history of receiving naloxone training, or their history of receiving overdose counseling exposure, which should be considered in future research. In this study, naloxone awareness served as a basic proxy for education, but future research should more deeply explore participants’ practical knowledge and usage. Five, we were unable to determine whether reported overdoses were intentional or unintentional and, we included PWUO and opioid dependence. Combining both types may limit our understanding, especially since opioid overdose and dependence intent are closely linked to suicidal ideation. Last, while the PHQ-9 provided an initial screening of suicidal ideation, future studies would benefit from using tools, such as the Columbia Suicide Severity Rating Scale and the Suicide Intent Scale, to capture more detailed data regarding suicide plans and attempts. There might be the potential for misclassification from PHQ-9 item 9 as a standalone indicator.

## Conclusion

Our study revealed a disproportionately high rate of suicidal ideation among PWUO. These findings underscore the need to integrate a suicidal risk screening tool within settings where PWUO are most likely to interact with healthcare settings, such as community-based SSPs, addiction treatment centers, and emergency departments, to facilitate early detection and linkage to mental health care within addiction treatment settings. Nearly one-third of participants in the study reported unmet mental health needs, emphasizing the urgent need for accessible, community-based interventions and targeted public health efforts. Interventions that address the shared causes and risk factors, such as programs to promote awareness of naloxone and expand access to mental health services for those with elevated depressive symptoms, are recommended.

## Supporting information

S1 Checklist STROBE checklist. (DOCX)
