# Supplementary material for: Suicidal ideation, mental health services, and barriers to care among syringe services program clients who use opioids
Source: PLoS One. 2026 Jul 30;21(7):e0354745. doi: 10.1371/journal.pone.0354745 (PMC13423042; doi:10.1371/journal.pone.0354745)
Supplement: Attachment — Submitted filename: Rebuttal_Letter.docx [file pone.0354745.s003.docx]

18 June 2026

Editor

PLOS One

Re: PONE-D-26-18198 “**Suicidal Ideation, Mental Health Services, and Barriers to Care Among Syringe Services Program Clients Who Use Opioids”**

Dear Editor Dr. Vincenzo De Luca

Thank you for your comments and the reviewer’s feedback regarding our manuscript titled **“**PONE-D-26-18198 “**Suicidal Ideation, Mental Health Services, and Barriers to Care Among Syringe Services Program Clients Who Use Opioids”** We have carefully revised the manuscript in response to the thorough and insightful observations we received. We are pleased to see interest in the topic and appreciate the constructive feedback.

We have addressed the comments point by point and have made corresponding changes in the manuscript in track changes (see below).

We hope this revised version will be suitable for publication in the PLOS One.

Sincerely,

Dr. Roman Shrestha, Ph.D., MPH

**Reviewer 1**

Recommendation to the editor: Minor revisions

Summary of the results: This cross-sectional study by Paudel et al. investigated the prevalence of suicidal ideation and barriers to accessing mental health services among people who use opioids at a syringe services program (N = 199) in New Haven. Results indicate that over one-third of people who use opioids reported recent suicidal ideation, with depressive symptoms strongly increasing risk and awareness of naloxone associated with lower risk. Despite high need, many participants faced barriers to mental health care, highlighting the importance of integrating suicide screening and improving access to services within such programs.

Overall impression:
This is a timely, well written and interesting study in an at-risk population group that is of interest for researchers and practitioners alike. I have some concerns regarding the methodology and results section which are listed below.

Introduction:

**#Comment 1:** Methods:
- Was the PHQ-8 (see line 154) or PHQ-9 used in this study? They differ regarding the suicidality item so I think PHQ-9 was used. Please specify.

**#Reply1:** Thank you for flagging this issue. This study used PHQ-9. Specifically, Item 9 of the PHQ-9 was used as the outcome variable to assess suicidal ideation, while the remaining eight items (PHQ-8) were used to measure depressive symptoms. We have explicitly clarified this in the Methods section.

**#Comment 2:** Results:
- Figure 2: Is there a table with the data used for Figure 2? I would like to know if these differences are statistically significant. If not please provide such table and indicate p-values or significance symbols in Figure 2.

**#Reply2:** Thank you so much for the suggestion, now we have added p value in the figure.

**#Comment 3:** In Table 1 and Table 2, sexual orientation was measured as a variable. Please add to the methods part how it was measured, why and what the authors make of the findings regarding sexual orientation.

**#Reply3:** Thank you for the suggestion now we have added this in the manuscript as

“Sexual orientation was assessed via a single self-report item. For analysis, responses were dichotomized into two categories: gay or lesbian and heterosexual.”

**#Comment 4:** Discussion:
- Please discuss the findings regarding sexual orientation

**#Reply4:** Thank you for this suggestion. Sexual orientation was not identified as a statistically significant risk factor for suicidal ideation in either the bivariate or multivariable analyses. Given this, and in the interest of maintaining a focused discussion centered on the study's primary findings, we have not included a dedicated discussion of sexual orientation. We appreciate the reviewer's perspective and acknowledge this which we could do in the future studies.

-**#Comment 5:** In the limitations section the authors discussed limitations regarding the low diversity of the sample (line 321). I was wondering what could be the strengths of the sample regarding sociodemographics (especially regarding sexual orientation and ethnicity)

**#Reply5:** Thank you for the suggestion. We have rewritten the strengths of the sample regarding the socio demographic, especially regarding sexual orientation and ethnicity as

“However, the sample included a substantial proportion of sexual minority individuals (gay or lesbian: 24.1%), considerably exceeding general population estimates, and meaningful representation of Hispanic (21.6%) which strengthens the relevance of findings for these underserved populations.”

**Reviewer #2:**

**#Comment 6:** Abstract
- Line 32 – define PWUO acronym again – when already introduced in line 27 – can just use acronym without typing out “people who use opioids

**#Reply6:** Thank you for flagging this issue, now we have made changes in the manuscript.

**#Comment 7:** Line 29-31 – is the gap with understanding suicidal risk AND its relation to risk? Or understanding suicidal risk itself? A little unclear and awkward sounding

**#Reply7:** Considering your suggestion, we made changes in the manuscript as:

“However, gaps persist in understanding suicidal risk among individuals attending SSPs and the extent to which such risk is associated with mental health service utilization in this setting.”

**#Comment 8:** Conclusion in abstract – demonstrates clinical importance well of work

**#Reply8:** Thank you for your appreciation.

**#Comment 9:** Introduction
- Line 67 – unclear what multi-level factors are (either define or explain how the examples listed are multi-level factors)

**#Reply9:** We thank the reviewer for this observation. The term "multi-level" was intended to convey that the contributing factors span individual-level clinical conditions (e.g., co-occurring mental health disorders, chronic pain, alcohol use disorder, opioid dependence, and overdose risk) and structural or social-level determinants (e.g., homelessness). We have revised the sentence to make this distinction explicit.

**#Comment 10:** 2nd paragraph and 3rd paragraph – would flesh out current link/evidence in PWUO and suicide risk – as 1st paragraph seems to be more of stats about how both are bad, but not necessarily how they’re connected (is there evidence that points to heightened suicide risk – especially suicidal ideation if that is relevant)

**#Reply10:** Thank you for the suggestion now we have added the paragraph and link evidence in PWUO and suicide risk. We have rewritten the introduction as given below to strength the introduction part of the manuscript.

A growing body of evidence underscores the interdependence between opioid use and suicidality. Individuals with opioid use are estimated to have suicide risk six times higher than the general populations [10]. Non-medical opioid use is consistently associated with increased odds of suicidal ideation [11]. Despite of high burden, they fall short in addressing the psychological and contextual factors such as housing instability, HIV risk, and inadequate access to mental health care that contribute to suicidality [12, 13].

**#Comment 11:** Methodology
- Line 99 – since you use term NHSSP later on, would put acronym after New Have SSP

**#Reply11:** Thank you for the suggestion, now we have made changes in the manuscript accordingly.

**#Comment 12:** Thought – any data on how many people did not fit this criteria from the New Haven SSP? Not needed but just interesting

**#Reply12:** All participants enrolled in the study met the eligibility criteria. Only individuals visiting the NHSP who met these criteria were referred to the Research Assistant for the study.

**#Comment 13:** Outcome variable – would talk about other studies that used the one item as a proxy – as I do have some methodological concerns about the psychometric validity of doing so. But if other studies have ESPECIALLY IF THEY DEMONSTRATED GOOD PSYCHOMETRIC PROPERTIES will help strengthen the case for this.

**#Reply13:** Although single-item measures are subject to psychometric limitations, PHQ-9 Item 9 has been widely used as a standalone proxy measure for suicidal ideation in research settings [Louzon 2016, Walker 2011, Bauer 2013]. Notably, validation studies have demonstrated acceptable sensitivity (87.6%) and high negative predictive value (97.2%) of this item against the Columbia Suicide Severity Rating Scale, supporting its use as a screening-level measure in population-based research.

<https://pure.johnshopkins.edu/en/publications/the-phq-9-item-9-based-screening-for-suicide-risk-a-validation-st/>

**#Comment 14:** Substance use and treatment related variables – since you talk about psychometrics of other established measures (e.g., PHQ-8) would do the same for the RODS

**#Reply14:** Thank you for the suggestion, now we have briefly talked about the psychometrics properties of the RODS in the manuscript. The RODS has demonstrated strong psychometric properties such as internal consistency α = 0.92, sensitivity (.97), and specificity (.76), as established by Wickersham, J. and et.al.

**#Comment 15:** Results
- No comments
**#Reply15:** Thank you for your review.

**#Comment 16:** Discussion
- Line 273 - once again – would be easier to interpret if muiltilevel factor was defined or operationalized (as it can be used inconsistently in literature)

**#Reply16:** Thank you for the suggestion, now we have already defined or operationalized it on the introduction section.

**#Comment 17:** Line 278-280 - would consider moving having at least 1-2 sentences to this effect in introduction to make connection between SI/suicide risk and PWUO more clear/importance more convincing – but could be other studies (if such ones exist)

**#Reply17:** Thank you for the suggestions, now we have added few sentences in the introduction and made connection between suicide and PWUO. Please see it in the revised manuscript.

**#Comment 18:** Good addressing 1 item for SI as a limitation

**#Reply18:** Thank you for the appreciation.

**#Comment 19:** Other/Overall Comments
- Using 1 item as measure of SI – checking in on current literature to build on case? – concern about this (See advice to mitigate)

**#Reply19:** Thank you for the suggestion. Despite the limitations of using, one item to measure suicidal ideation, there are scientific articles who have used PHQ-9 , 9^th^ item to measure suicidal ideation. Here are few:

1. Liu, Z., Zhang, Y. & Li, Q. Two-week prevalence and correlates of suicidal ideation among first-visit general-hospital psychiatric outpatients: a cross-sectional study using the PHQ-9 and EPQ. *BMC Psychiatry* **26**, 320 (2026). <https://doi.org/10.1186/s12888-026-07960-y>
2. Louzon, S. A., Bossarte, R., McCarthy, J. F., & Katz, I. R. (2016). The Clinical Utility of PHQ-9 Item 9 for Suicide Prediction: In Reply. *Psychiatric services (Washington, D.C.)*, *67*(9), 1042–1043. <https://doi.org/10.1176/appi.ps.670903>
3. Yarborough, B. J. H., Stumbo, S. P., Ahmedani, B., Rossom, R., Coleman, K., Boggs, J. M., & Simon, G. E. (2021). Suicide Behavior Following PHQ-9 Screening Among Individuals With Substance Use Disorders. *Journal of addiction medicine*, *15*(1), 55–60. <https://doi.org/10.1097/ADM.0000000000000696>
4. Kim, S., Lee, H. K., & Lee, K. (2021). Which PHQ-9 Items Can Effectively Screen for Suicide? Machine Learning Approaches. *International journal of environmental research and public health*, *18*(7), 3339. <https://doi.org/10.3390/ijerph18073339>
5. Liu, H., Dong, H., Jin, M., Zhou, Y., Hao, H., Yuan, Y., Jia, H., & Li, J. (2025). Association between overactive bladder and suicidal ideation in US adults: a population-based study. *Frontiers in psychiatry*, *16*, 1483684. <https://doi.org/10.3389/fpsyt.2025.1483684>

**#Comment 20:** However clinical importance explained well in abstract and discussion (though needs to make connection between suicide risk and PWUO more clear in introduction)

**#Reply20:** Thank you for the suggestion, now we have made changes in the revised manuscript.
