# Supplementary material for: Suicidal ideation, mental health services, and barriers to care among syringe services program clients who use opioids
Source: PLoS One. 2026 Jul 30;21(7):e0354745. doi: 10.1371/journal.pone.0354745 (PMC13423042; doi:10.1371/journal.pone.0354745)
Supplement: Peer review history

# Peer review: Decision Letter 0

**DOI:** 10.1371/journal.pone.0354745.r001

**Authors:** Vincenzo De Luca

3 Jun 2026

PONE-D-26-18198

Suicidal Ideation, Mental Health Services, and Barriers to Care Among Syringe Services Program Clients Who Use Opioids

PLOS One

Dear Dr. Shrestha,

Thank you for submitting your manuscript to PLOS ONE. After careful consideration, we feel that it has merit but does not fully meet PLOS ONE’s publication criteria as it currently stands. Therefore, we invite you to submit a revised version of the manuscript that addresses the points raised during the review process.

Please submit your revised manuscript by Jul 15 2026 11:59PM. If you will need more time than this to complete your revisions, please reply to this message or contact the journal office at plosone@plos.org. When you're ready to submit your revision, log on to https://www.editorialmanager.com/pone/ and select the 'Submissions Needing Revision' folder to locate your manuscript file.

Please include the following items when submitting your revised manuscript:

- A letter that responds to each point raised by the academic editor and reviewer(s). You should upload this letter as a separate file labeled 'Response to Reviewers'.
- A marked-up copy of your manuscript that highlights changes made to the original version. You should upload this as a separate file labeled 'Revised Manuscript with Track Changes'.
- An unmarked version of your revised paper without tracked changes. You should upload this as a separate file labeled 'Manuscript'.

If you would like to make changes to your financial disclosure, please include your updated statement in your cover letter. Guidelines for resubmitting your figure files are available below the reviewer comments at the end of this letter.

If applicable, we recommend that you deposit your laboratory protocols in protocols.io to enhance the reproducibility of your results. Protocols.io assigns your protocol its own identifier (DOI) so that it can be cited independently in the future. For instructions see: https://journals.plos.org/plosone/s/submission-guidelines#loc-laboratory-protocols. Additionally, PLOS ONE offers an option for publishing peer-reviewed Lab Protocol articles, which describe protocols hosted on protocols.io. Read more information on sharing protocols at https://plos.org/protocols?utm_medium=editorial-email&utm_source=authorletters&utm_campaign=protocols.

As the corresponding author, your ORCID iD is verified in the submission system and will appear in the published article. PLOS supports the use of ORCID, and we encourage all coauthors to register for an ORCID iD and use it as well. Please encourage your coauthors to verify their ORCID iD within the submission system before final acceptance, as unverified ORCID iDs will not appear in the published article. Only the individual author can complete the verification step; PLOS staff cannot verify ORCID iDs on behalf of authors.

We look forward to receiving your revised manuscript.

Kind regards,

Vincenzo De Luca

Academic Editor

PLOS One

Journal Requirements:

When submitting your revision, we need you to address these additional requirements.

1. Please ensure that your manuscript meets PLOS ONE's style requirements, including those for file naming. The PLOS ONE style templates can be found at

https://journals.plos.org/plosone/s/file?id=wjVg/PLOSOne_formatting_sample_main_body.pdf and

https://journals.plos.org/plosone/s/file?id=ba62/PLOSOne_formatting_sample_title_authors_affiliations.pdf

2. Please include your full ethics statement in the ‘Methods’ section of your manuscript file. In your statement, please include the full name of the IRB or ethics committee who approved or waived your study, as well as whether or not you obtained informed written or verbal consent. If consent was waived for your study, please include this information in your statement as well.

3.  In this instance it seems there may be acceptable restrictions in place that prevent the public sharing of your minimal data. However, in line with our goal of ensuring long-term data availability to all interested researchers, PLOS’ Data Policy states that authors cannot be the sole named individuals responsible for ensuring data access (http://journals.plos.org/plosone/s/data-availability#loc-acceptable-data-sharing-methods).

Data requests to a non-author institutional point of contact, such as a data access or ethics committee, helps guarantee long term stability and availability of data. Providing interested researchers with a durable point of contact ensures data will be accessible even if an author changes email addresses, institutions, or becomes unavailable to answer requests.

Before we proceed with your manuscript, please also provide non-author contact information (phone/email/hyperlink) for a data access committee, ethics committee, or other institutional body to which data requests may be sent. If no institutional body is available to respond to requests for your minimal data, please consider if there any institutional representatives who did not collaborate in the study, and are not listed as authors on the manuscript, who would be able to hold the data and respond to external requests for data access? If so, please provide their contact information (i.e., email address). Please also provide details on how you will ensure persistent or long-term data storage and availability.

4. Please include captions for your Supporting Information files at the end of your manuscript, and update any in-text citations to match accordingly. Please see our Supporting Information guidelines for more information: http://journals.plos.org/plosone/s/supporting-information.

5. If the reviewer comments include a recommendation to cite specific previously published works, please review and evaluate these publications to determine whether they are relevant and should be cited. There is no requirement to cite these works unless the editor has indicated otherwise.

[Note: HTML markup is below. Please do not edit.]

Reviewers' comments:

Reviewer's Responses to Questions

**Comments to the Author**

1. Is the manuscript technically sound, and do the data support the conclusions?

The manuscript must describe a technically sound piece of scientific research with data that supports the conclusions. Experiments must have been conducted rigorously, with appropriate controls, replication, and sample sizes. The conclusions must be drawn appropriately based on the data presented.

Reviewer #1: Yes

Reviewer #2: Partly

**********

2. Has the statistical analysis been performed appropriately and rigorously?

Reviewer #1: Yes

Reviewer #2: Yes

**********

3. Have the authors made all data underlying the findings in their manuscript fully available?

The PLOS Data policy requires authors to make all data underlying the findings described in their manuscript fully available without restriction, with rare exception (please refer to the Data Availability Statement in the manuscript PDF file). The data should be provided as part of the manuscript or its supporting information, or deposited to a public repository. For example, in addition to summary statistics, the data points behind means, medians and variance measures should be available. If there are restrictions on publicly sharing data—e.g. participant privacy or use of data from a third party—those must be specified.

Reviewer #1: Yes

Reviewer #2: Yes

**********

4. Is the manuscript presented in an intelligible fashion and written in standard English?

PLOS ONE does not copyedit accepted manuscripts, so the language in submitted articles must be clear, correct, and unambiguous. Any typographical or grammatical errors should be corrected at revision, so please note any specific errors here.

Reviewer #1: Yes

Reviewer #2: Yes

**********

5. Review Comments to the Author

Please use the space provided to explain your answers to the questions above. You may also include additional comments for the author, including concerns about dual publication, research ethics, or publication ethics. (Please upload your review as an attachment if it exceeds 20,000 characters)

Reviewer #1: Review Template PLOS One

Recommendation to the editor: Minor revisions

Summary of the results: This cross-sectional study by Paudel et al. investigated the prevalence of suicidal ideation and barriers to accessing mental health services among people who use opioids at a syringe services program (N = 199) in New Haven. Results indicate that over one-third of people who use opioids reported recent suicidal ideation, with depressive symptoms strongly increasing risk and awareness of naloxone associated with lower risk. Despite high need, many participants faced barriers to mental health care, highlighting the importance of integrating suicide screening and improving access to services within such programs.

Overall impression:

This is a timely, well written and interesting study in an at-risk population group that is of interest for researchers and practitioners alike. I have some concerns regarding the methodology and results section which are listed down below.

Introduction:

Methods:

- Was the PHQ-8 (see line 154) or PHQ-9 used in this study? They differ regarding the suicidality item so I think PHQ-9 was used. Please specify.

Results:

- Figure 2: Is there a table with the data used for Figure 2? I would like to know if these differences are statistically significant. If not please provide such table and indicate p-values or significance symbols in Figure 2.

- In Table 1 and Table 2, sexual orientation was measured as a variable. Please add to the methods part how it was measured, why and what the authors make of the findings regarding sexual orientation.

Discussion:

- Please discuss the findings regarding sexual orientation

- In the limitations section the authors discussed limitations regarding the low diversity of the sample (line 321). I was wondering what could be the strengths of the sample regarding sociodemographics (especially regarding sexual orientation and ethnicity)

Criteria for Publication

1. The study presents the results of original research.

YES

2. Results reported have not been published elsewhere.

YES

3. Experiments, statistics, and other analyses are performed to a high technical standard and are described in sufficient detail.

PARTIALLY YES

4. Conclusions are presented in an appropriate fashion and are supported by the data.

PARTIALLY YES

5. The article is presented in an intelligible fashion and is written in standard English.

YES

6. The research meets all applicable standards for the ethics of experimentation and research integrity.

YES

7. The article adheres to appropriate reporting guidelines and community standards for data availability.

YES

Reviewer #2: Abstract

- Line 32 – define PWUO acronym again – when already introduced in line 27 – can just use acronym without typing out “people who use opioids

- Line 29-31 – is the gap with understanding suicidal risk AND its relation to risk? Or understanding suicidal risk itself? A little unclear and awkward sounding

- Conclusion in abstract – demonstrates clinical importance well of work

Introduction

- Line 67 – unclear what multi-level factors are (either define or explain how the examples listed are multi-level factors)

- 2nd paragraph and 3rd paragraph – would flesh out current link/evidence in PWUO and suicide risk – as 1st paragraph seems to be more of stats about how both are bad, but not necessarily how they’re connected (is there evidence that points to heightened suicide risk – especially suicidal ideation if that is relevant)

Methodology

- Line 99 – since you use term NHSSP later on, would put acronym after New Have SSP

- Thought – any data on how many people did not fit this criteria from the New Haven SSP? Not needed but just interesting

- Outcome variable – would talk about other studies that used the one item as a proxy – as I do have some methodological concerns about the psychometric validity of doing so. But if other studies have ESPECIALLY IF THEY DEMONSTRATED GOOD PSYCHOMETRIC PROPERTIES will help strengthen the case for this.

- Substance use and treatment related variables – since you talk about psychometrics of other established measures (e.g., PHQ-8) would do the same for the RODS

Results

- No comments

Discussion

- Line 273 - once again – would be easier to interpret if muiltilevel factor was defined or operationalized (as it can be used inconsistently in literature)

- Line 278-280 - would consider moving having at least 1-2 sentences to this effect in introduction to make connection between SI/suicide risk and PWUO more clear/importance more convincing – but could be other studies (if such ones exist)

- Good addressing 1 item for SI as a limitation

Other/Overall Comments

- Using 1 item as measure of SI – checking in on current literature to build on case? – concern about this (See advice to mitigate)

- However clinical importance explained well in abstract and discussion (though needs to make connection between suicide risk and PWUO more clear in introduction)

**********

6. PLOS authors have the option to publish the peer review history of their article (what does this mean?). If published, this will include your full peer review and any attached files.

If you choose “no”, your identity will remain anonymous but your review may still be made public.

**Do you want your identity to be public for this peer review?**  For information about this choice, including consent withdrawal, please see our Privacy Policy.

Reviewer #1: No

Reviewer #2: No

**********

[NOTE: If reviewer comments were submitted as an attachment file, they will be attached to this email and accessible via the submission site. Please log into your account, locate the manuscript record, and check for the action link "View Attachments". If this link does not appear, there are no attachment files.]

To ensure your figures meet our technical requirements, please review our figure guidelines: https://journals.plos.org/plosone/s/figures

You may also use PLOS’s free figure tool, NAAS, to help you prepare publication quality figures: https://journals.plos.org/plosone/s/figures#loc-tools-for-figure-preparation.

NAAS will assess whether your figures meet our technical requirements by comparing each figure against our figure specifications.

# Author comment: Author response to Decision Letter 1

**DOI:** 10.1371/journal.pone.0354745.r002

30 Jun 2026

18 June 2026

Editor

PLOS One

Re: PONE-D-26-18198 “Suicidal Ideation, Mental Health Services, and Barriers to Care Among Syringe Services Program Clients Who Use Opioids”

Dear Editor Dr. Vincenzo De Luca

Thank you for your comments and the reviewer’s feedback regarding our manuscript titled “PONE-D-26-18198 “Suicidal Ideation, Mental Health Services, and Barriers to Care Among Syringe Services Program Clients Who Use Opioids” We have carefully revised the manuscript in response to the thorough and insightful observations we received. We are pleased to see interest in the topic and appreciate the constructive feedback.

We have addressed the comments point by point and have made corresponding changes in the manuscript in track changes (see below).

We hope this revised version will be suitable for publication in the PLOS One.

Sincerely,

Dr. Roman Shrestha, Ph.D., MPH

Reviewer 1

Recommendation to the editor: Minor revisions

Summary of the results: This cross-sectional study by Paudel et al. investigated the prevalence of suicidal ideation and barriers to accessing mental health services among people who use opioids at a syringe services program (N = 199) in New Haven. Results indicate that over one-third of people who use opioids reported recent suicidal ideation, with depressive symptoms strongly increasing risk and awareness of naloxone associated with lower risk. Despite high need, many participants faced barriers to mental health care, highlighting the importance of integrating suicide screening and improving access to services within such programs.

Overall impression:

This is a timely, well written and interesting study in an at-risk population group that is of interest for researchers and practitioners alike. I have some concerns regarding the methodology and results section which are listed below.

Introduction:

#Comment 1: Methods:

- Was the PHQ-8 (see line 154) or PHQ-9 used in this study? They differ regarding the suicidality item so I think PHQ-9 was used. Please specify.

#Reply1: Thank you for flagging this issue. This study used PHQ-9. Specifically, Item 9 of the PHQ-9 was used as the outcome variable to assess suicidal ideation, while the remaining eight items (PHQ-8) were used to measure depressive symptoms. We have explicitly clarified this in the Methods section.

#Comment 2: Results:

- Figure 2: Is there a table with the data used for Figure 2? I would like to know if these differences are statistically significant. If not please provide such table and indicate p-values or significance symbols in Figure 2.

#Reply2: Thank you so much for the suggestion, now we have added p value in the figure.

#Comment 3: In Table 1 and Table 2, sexual orientation was measured as a variable. Please add to the methods part how it was measured, why and what the authors make of the findings regarding sexual orientation.

#Reply3: Thank you for the suggestion now we have added this in the manuscript as

“Sexual orientation was assessed via a single self-report item. For analysis, responses were dichotomized into two categories: gay or lesbian and heterosexual.”

#Comment 4: Discussion:

- Please discuss the findings regarding sexual orientation

#Reply4: Thank you for this suggestion. Sexual orientation was not identified as a statistically significant risk factor for suicidal ideation in either the bivariate or multivariable analyses. Given this, and in the interest of maintaining a focused discussion centered on the study's primary findings, we have not included a dedicated discussion of sexual orientation. We appreciate the reviewer's perspective and acknowledge this which we could do in the future studies.

-#Comment 5: In the limitations section the authors discussed limitations regarding the low diversity of the sample (line 321). I was wondering what could be the strengths of the sample regarding sociodemographics (especially regarding sexual orientation and ethnicity)

#Reply5: Thank you for the suggestion. We have rewritten the strengths of the sample regarding the socio demographic, especially regarding sexual orientation and ethnicity as

“However, the sample included a substantial proportion of sexual minority individuals (gay or lesbian: 24.1%), considerably exceeding general population estimates, and meaningful representation of Hispanic (21.6%) which strengthens the relevance of findings for these underserved populations.”

Reviewer #2:

#Comment 6: Abstract

- Line 32 – define PWUO acronym again – when already introduced in line 27 – can just use acronym without typing out “people who use opioids

#Reply6: Thank you for flagging this issue, now we have made changes in the manuscript.

#Comment 7: Line 29-31 – is the gap with understanding suicidal risk AND its relation to risk? Or understanding suicidal risk itself? A little unclear and awkward sounding

#Reply7: Considering your suggestion, we made changes in the manuscript as:

“However, gaps persist in understanding suicidal risk among individuals attending SSPs and the extent to which such risk is associated with mental health service utilization in this setting.”

#Comment 8: Conclusion in abstract – demonstrates clinical importance well of work

#Reply8: Thank you for your appreciation.

#Comment 9: Introduction

- Line 67 – unclear what multi-level factors are (either define or explain how the examples listed are multi-level factors)

#Reply9: We thank the reviewer for this observation. The term "multi-level" was intended to convey that the contributing factors span individual-level clinical conditions (e.g., co-occurring mental health disorders, chronic pain, alcohol use disorder, opioid dependence, and overdose risk) and structural or social-level determinants (e.g., homelessness). We have revised the sentence to make this distinction explicit.

#Comment 10: 2nd paragraph and 3rd paragraph – would flesh out current link/evidence in PWUO and suicide risk – as 1st paragraph seems to be more of stats about how both are bad, but not necessarily how they’re connected (is there evidence that points to heightened suicide risk – especially suicidal ideation if that is relevant)

#Reply10: Thank you for the suggestion now we have added the paragraph and link evidence in PWUO and suicide risk. We have rewritten the introduction as given below to strength the introduction part of the manuscript.

A growing body of evidence underscores the interdependence between opioid use and suicidality. Individuals with opioid use are estimated to have suicide risk six times higher than the general populations [10]. Non-medical opioid use is consistently associated with increased odds of suicidal ideation [11]. Despite of high burden, they fall short in addressing the psychological and contextual factors such as housing instability, HIV risk, and inadequate access to mental health care that contribute to suicidality [12, 13].

#Comment 11: Methodology

- Line 99 – since you use term NHSSP later on, would put acronym after New Have SSP

#Reply11: Thank you for the suggestion, now we have made changes in the manuscript accordingly.

#Comment 12: Thought – any data on how many people did not fit this criteria from the New Haven SSP? Not needed but just interesting

#Reply12: All participants enrolled in the study met the eligibility criteria. Only individuals visiting the NHSP who met these criteria were referred to the Research Assistant for the study.

#Comment 13: Outcome variable – would talk about other studies that used the one item as a proxy – as I do have some methodological concerns about the psychometric validity of doing so. But if other studies have ESPECIALLY IF THEY DEMONSTRATED GOOD PSYCHOMETRIC PROPERTIES will help strengthen the case for this.

#Reply13: Although single-item measures are subject to psychometric limitations, PHQ-9 Item 9 has been widely used as a standalone proxy measure for suicidal ideation in research settings [Louzon 2016, Walker 2011, Bauer 2013]. Notably, validation studies have demonstrated acceptable sensitivity (87.6%) and high negative predictive value (97.2%) of this item against the Columbia Suicide Severity Rating Scale, supporting its use as a screening-level measure in population-based research.

https://pure.johnshopkins.edu/en/publications/the-phq-9-item-9-based-screening-for-suicide-risk-a-validation-st/

#Comment 14: Substance use and treatment related variables – since you talk about psychometrics of other established measures (e.g., PHQ-8) would do the same for the RODS

#Reply14: Thank you for the suggestion, now we have briefly talked about the psychometrics properties of the RODS in the manuscript. The RODS has demonstrated strong psychometric properties such as internal consistency α = 0.92, sensitivity (.97), and specificity (.76), as established by Wickersham, J. and et.al.

#Comment 15: Results

- No comments

#Reply15: Thank you for your review.

#Comment 16: Discussion

- Line 273 - once again – would be easier to interpret if muiltilevel factor was defined or operationalized (as it can be used inconsistently in literature)

#Reply16: Thank you for the suggestion, now we have already defined or operationalized it on the introduction section.

#Comment 17: Line 278-280 - would consider moving having at least 1-2 sentences to this effect in introduction to make connection between SI/suicide risk and PWUO more clear/importance more convincing – but could be other studies (if such ones exist)

#Reply17: Thank you for the suggestions, now we have added few sentences in the introduction and made connection between suicide and PWUO. Please see it in the revised manuscript.

#Comment 18: Good addressing 1 item for SI as a limitation

#Reply18: Thank you for the appreciation.

#Comment 19: Other/Overall Comments

- Using 1 item as measure of SI – checking in on current literature to build on case? – concern about this (See advice to mitigate)

#Reply19: Thank you for the suggestion. Despite the limitations of using, one item to measure suicidal ideation, there are scientific articles who have used PHQ-9 , 9th item to measure suicidal ideation. Here are few:

1. Liu, Z., Zhang, Y. & Li, Q. Two-week prevalence and correlates of suicidal ideation among first-visit general-hospital psychiatric outpatients: a cross-sectional study using the PHQ-9 and EPQ. BMC Psychiatry 26, 320 (2026). https://doi.org/10.1186/s12888-026-07960-y

2. Louzon, S. A., Bossarte, R., McCarthy, J. F., & Katz, I. R. (2016). The Clinical Utility of PHQ-9 Item 9 for Suicide Prediction: In Reply. Psychiatric services (Washington, D.C.), 67(9), 1042–1043. https://doi.org/10.1176/appi.ps.670903

3. Yarborough, B. J. H., Stumbo, S. P., Ahmedani, B., Rossom, R., Coleman, K., Boggs, J. M., & Simon, G. E. (2021). Suicide Behavior Following PHQ-9 Screening Among Individuals With Substance Use Disorders. Journal of addiction medicine, 15(1), 55–60. https://doi.org/10.1097/ADM.0000000000000696

4. Kim, S., Lee, H. K., & Lee, K. (2021). Which PHQ-9 Items Can Effectively Screen for Suicide? Machine Learning Approaches. International journal of environmental research and public health, 18(7), 3339. https://doi.org/10.3390/ijerph18073339

5. Liu, H., Dong, H., Jin, M., Zhou, Y., Hao, H., Yuan, Y., Jia, H., & Li, J. (2025). Association between overactive bladder and suicidal ideation in US adults: a population-based study. Frontiers in psychiatry, 16, 1483684. https://doi.org/10.3389/fpsyt.2025.1483684

#Comment 20: However clinical importance explained well in abstract and discussion (though needs to make connection between suicide risk and PWUO more clear in introduction)

#Reply20: Thank you for the suggestion, now we have made changes in the revised manuscript.

Attachment Submitted filename: Rebuttal_Letter.docx

# Peer review: Decision Letter 1

**DOI:** 10.1371/journal.pone.0354745.r003

**Authors:** Vincenzo De Luca

13 Jul 2026

Suicidal Ideation, Mental Health Services, and Barriers to Care Among Syringe Services Program Clients Who Use Opioids

PONE-D-26-18198R1

Dear Dr. Shrestha,

We’re pleased to inform you that your manuscript has been judged scientifically suitable for publication and will be formally accepted for publication once it meets all outstanding technical requirements.

Within one week, you’ll receive an e-mail detailing the required amendments. When these have been addressed, you’ll receive a formal acceptance letter and your manuscript will be scheduled for publication.

An invoice will be generated when your article is formally accepted. Please note, if your institution has a publishing partnership with PLOS and your article meets the relevant criteria, all or part of your publication costs will be covered. Please make sure your user information is up-to-date by logging into Editorial Manager at Editorial Manager® and clicking the ‘Update My Information' link at the top of the page. For questions related to billing, please contact billing support.

If your institution or institutions have a press office, please notify them about your upcoming paper to help maximize its impact. If they’ll be preparing press materials, please inform our press team as soon as possible -- no later than 48 hours after receiving the formal acceptance. Your manuscript will remain under strict press embargo until 2 pm Eastern Time on the date of publication. For more information, please contact onepress@plos.org.

Kind regards,

Vincenzo De Luca

Academic Editor

PLOS One

Additional Editor Comments (optional):

Reviewers' comments:

Reviewer's Responses to Questions

**Comments to the Author**

1. If the authors have adequately addressed your comments raised in a previous round of review and you feel that this manuscript is now acceptable for publication, you may indicate that here to bypass the “Comments to the Author” section, enter your conflict of interest statement in the “Confidential to Editor” section, and submit your "Accept" recommendation.

Reviewer #1: All comments have been addressed

**********

2. Is the manuscript technically sound, and do the data support the conclusions?

The manuscript must describe a technically sound piece of scientific research with data that supports the conclusions. Experiments must have been conducted rigorously, with appropriate controls, replication, and sample sizes. The conclusions must be drawn appropriately based on the data presented.

Reviewer #1: Yes

**********

3. Has the statistical analysis been performed appropriately and rigorously?

Reviewer #1: Yes

**********

4. Have the authors made all data underlying the findings in their manuscript fully available?

The PLOS Data policy requires authors to make all data underlying the findings described in their manuscript fully available without restriction, with rare exception (please refer to the Data Availability Statement in the manuscript PDF file). The data should be provided as part of the manuscript or its supporting information, or deposited to a public repository. For example, in addition to summary statistics, the data points behind means, medians and variance measures should be available. If there are restrictions on publicly sharing data—e.g. participant privacy or use of data from a third party—those must be specified.

Reviewer #1: Yes

**********

5. Is the manuscript presented in an intelligible fashion and written in standard English?

PLOS ONE does not copyedit accepted manuscripts, so the language in submitted articles must be clear, correct, and unambiguous. Any typographical or grammatical errors should be corrected at revision, so please note any specific errors here.

Reviewer #1: Yes

**********

6. Review Comments to the Author

Please use the space provided to explain your answers to the questions above. You may also include additional comments for the author, including concerns about dual publication, research ethics, or publication ethics. (Please upload your review as an attachment if it exceeds 20,000 characters)

Reviewer #1: Thank you for implementing my comments and suggestions. I have no further concerns regarding the manuscript.

**********

7. PLOS authors have the option to publish the peer review history of their article (what does this mean?). If published, this will include your full peer review and any attached files.

If you choose “no”, your identity will remain anonymous but your review may still be made public.

**Do you want your identity to be public for this peer review?**  For information about this choice, including consent withdrawal, please see our Privacy Policy.

Reviewer #1: No

**********

# Editor report: Acceptance letter

**DOI:** 10.1371/journal.pone.0354745.r004

**Authors:** Vincenzo De Luca

PONE-D-26-18198R1

PLOS One

Dear Dr. Shrestha,

I'm pleased to inform you that your manuscript has been deemed suitable for publication in PLOS One. Congratulations! Your manuscript is now being handed over to our production team.

At this stage, our production department will prepare your paper for publication. This includes ensuring the following:

* All references, tables, and figures are properly cited

* All relevant supporting information is included in the manuscript submission,

* There are no issues that prevent the paper from being properly typeset

You will receive further instructions from the production team, including instructions on how to review your proof when it is ready. Please keep in mind that we are working through a large volume of accepted articles, so please give us a few days to review your paper and let you know the next and final steps.

Lastly, if your institution or institutions have a press office, please let them know about your upcoming paper now to help maximize its impact. If they'll be preparing press materials, please inform our press team within the next 48 hours. Your manuscript will remain under strict press embargo until 2 pm Eastern Time on the date of publication. For more information, please contact onepress@plos.org.

You will receive an invoice from PLOS for your publication fee after your manuscript has reached the completed accept phase. If you receive an email requesting payment before acceptance or for any other service, this may be a phishing scheme. Learn how to identify phishing emails and protect your accounts at https://explore.plos.org/phishing.

If we can help with anything else, please email us at customercare@plos.org.

Thank you for submitting your work to PLOS ONE and supporting open access.

Kind regards,

PLOS ONE Editorial Office Staff

on behalf of

Dr. Vincenzo De Luca

Academic Editor

PLOS One
